# hTERT-Immortalized Bone Mesenchymal Stromal Cells Expressing Rat Galanin via a Single Tetracycline-Inducible Lentivirus System

**DOI:** 10.1155/2017/6082684

**Published:** 2017-05-11

**Authors:** Ke An, Hui-ping Liu, Xiao-long Zhong, David Y. B. Deng, Jing-jun Zhang, Zhi-heng Liu

**Affiliations:** ^1^Department of Anesthesiology, The First Affiliated Hospital of Sun Yat-sen University, Guangzhou 510080, China; ^2^Department of Anesthesiology, The Third Xiangya Hospital of Central South University, Changsha 410013, China; ^3^Laboratory of Research Center for Translational Medicine, The First Affiliated Hospital of Sun Yat-sen University, Guangzhou 510080, China; ^4^Department of Anesthesiology, The First Affiliated Hospital of Shenzhen University, Shenzhen 518035, China

## Abstract

The use of human telomerase reverse transcriptase-immortalized bone marrow mesenchymal stromal cells (hTERT-BMSCs) as vehicles to deliver antinociceptive galanin (GAL) molecules into pain-processing centers represents a novel cell therapy strategy for pain management. Here, an hTERT-BMSCs/Tet-on/GAL cell line was constructed using a single Tet-on-inducible lentivirus system, and subsequent experiments demonstrated that the secretion of rat GAL from hTERT-BMSCs/Tet-on/GAL was switched on and off under the control of an inducer in a dose-dependent manner. The construction of this cell line is the first promising step in the regulation of GAL secretion from hTERT-immortalized BMSCs, and the potential application of this system may provide a stem cell-based research platform for pain.

## 1. Introduction

Treatment of chronic neuropathic pain resulting from peripheral nerve injury is one of the most difficult problems in modern clinical practice. Although current treatments, such as traditional pharmacological approaches, are often effective for limited periods, these therapies have no practical significance for the progression of pain and can even induce tolerance and unacceptable systemic side effects. Diminished inhibitory neurotransmission in the superficial dorsal horn, particularly when there is an imbalance of excitatory and inhibitory systems, is the likely mechanism underlying the induction and development of neuropathic pain following nerve injury [1, 2]. Therefore, alternative methods targeting mechanisms of neuropathic pain are needed.

The use of cell lines as “biological minipumps” to chronically deliver antinociceptive molecules into the pain-processing centers of the spinal cord represents a newly developed technique for the treatment of pain [3]. Galanin (GAL) is a neuropeptide of 29 or 30 (in humans) amino acids that is proteolytically processed from the peptide precursor preprogalanin. GAL is widely distributed throughout the central and peripheral nervous system and is involved in a variety of physiological and pathophysiological activities, including pain signaling [4]. Extensive research has demonstrated that this molecule plays a gatekeeper role in the inhibition of neuropathic pain [5, 6]. Previous studies have demonstrated that immortalized astrocytes are not only easily manipulated, reproducible, and nontumorigenic but are also safe potential vehicles for the delivery of therapeutic genes (galanin) for chronic pain therapy [7–9].

However, obtaining primary neuronal cells from adult tissue is difficult and faces major ethical issues in clinical practice. Studies have increasingly focused on the potential therapeutic effects of stem cell transplantation for neurological diseases [10]. Bone marrow stem cells, including the pluripotent hematopoietic stem cells (HSCs) and bone mesenchymal stem cells (BMSCs), are being considered as potential targets for cell and gene therapy-based approaches against a variety of different diseases. Although human HSCs as vehicles to treat metachromatic leukodystrophy (MLD) has been used to treat patients with early onset MLD in a phase I/II trial, the HSCs give rise to all different blood cell lineages, such as the myeloid and lymphoid cell lineages [11]. In contrast, BMSCs are capable of differentiating into mesenchymal lineages such as osteoblasts, chondrocytes, adipocytes, and even neurons and astrocytes [12]. BMSCs can also be engineered to secrete a variety of different proteins in vitro and in vivo that could potentially treat a variety of serum protein deficiencies and other genetic or acquired diseases [13]. Indeed, the potent pathotropic migratory properties of BMSCs and ability to circumvent both the complications associated with immune rejection of allogenic cells and many of the moral reasons associated with embryonic stem cell use suggest that BMSCs are most promising stem cells as a potential target for the clinical use of genetically engineered stem cells [14, 15]. However, BMSCs have a low proliferative ability with a finite lifespan in vitro; this limitation has been overcome via ectopic expression of human telomerase reverse transcriptase (hTERT), the catalytic component of telomerase, to produce large quantities of these cells as an attractive source for cellular transplantation [16–18].

The ability to switch on and off the expression of transgenes delivered via lentiviral vectors is desirable in a number of experimental and therapeutic situations in which the transgene product must be regulated in a timely manner. An ideal lentiviral-based system should be contained within a single vector to avoid the need for multiple transductions of the target cells with high multiplicities of infection (MOI), which would increase the risk of insertional mutagenesis [19]. The most widely studied system for gene regulation in eukaryotic cells is the tetracycline- (Tet-) regulated transgene expression system, which employs a Tn*10* Tet resistance operator derived from *Escherichia coli* [20]. The Tet-inducible system has been extensively used to control transgene expression in stem cells. Therefore, to enhance the consistent and controllable exogenous expression of the GAL gene, a new stem cell-based approach was developed by transfecting a single inducible Tet-On lentiviral vector- (LV-) mediated GAL gene delivery system into hTERT-immortalized BMSCs. We hypothesized that these newly developed stem cells will serve as efficient and controllable pools for GAL expression within the CNS for further pain study.

## 2. Materials and Methods

See supplemental information available online at https://doi.org/10.1155/2017/6082684 for detailed descriptions.

### 2.1. Ethic Statement

All procedures were conducted in accordance with the Ethical Guidelines of the International Association for the Study of Pain (1983) and approved by the Administrative Committee of Experimental Animal Care and Use of Sun Yat-sen University (permit number: 2013-A-001).

### 2.2. Lentiviral Vector Construction and Production

**Figure 1:**
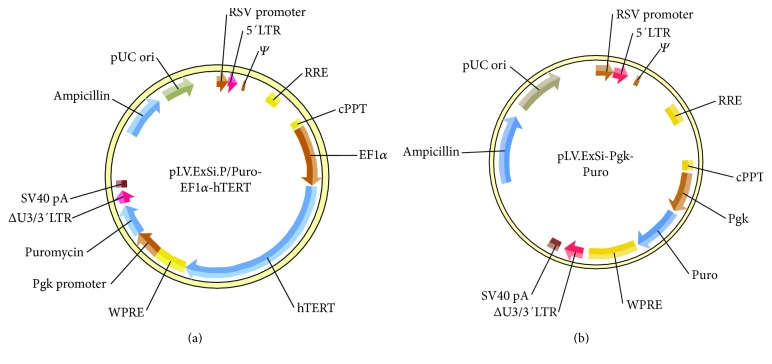
Plasmid profiles of the pLV.ExSi/Puro-EF1*α*-hTERT (a) and pLV.ExSi/ PGK-Puro (b) vectors with and without the hTERT gene, respectively, used for the immortalization of primary rat BMSCs (Cyagen Biosciences).

**Figure 2:**
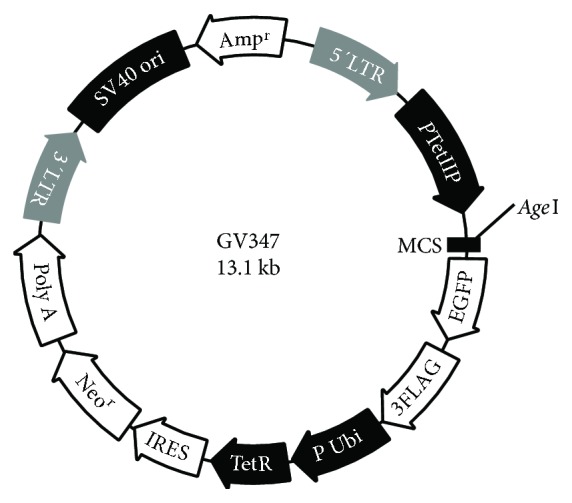
Plasmid profile of the single tetracycline-inducible lentiviral backbone vector system pLV.TetIIP-EGFP-/Ubi-TetR (Genechem, GV347). The system comprises an EGFP reporter gene and a tetracycline (Tet) response element under the control of separate promoters, the TetIIP and Ubi promoters. This construct drives the expression of rat galanin from transduced cells via Dox induction after cloning of the GAL gene into the multiple cloning sites (MCS).

The plasmid pCI-Neo-hTERT containing hTERT cDNA was kindly provided by Professor William C. Hahn (Dana-Farber Cancer Institute, Harvard Medical School, USA). Using Gateway® Recombination Cloning Technology [21], the specific fragments of EF1*α*-hTERT containing the attB adaptor were PCR amplified using Phusion® high-fidelity DNA polymerase (New England BioLabs, Singapore). In the subsequent BP recombination reaction (between attB and attP sites), the PCR product containing attB was transferred to a kanamycin-resistant donor vector (pENTR). Finally, the lentiviral vector pLV.ExSi.P/Puro-EF1*α*-hTERT was constructed after cloning the EF1*α*-hTERT-specific gene into pLV.ExSi.P/Pgk-Puro expression vectors via an LR recombination reaction (between attL and attR sites) (Cyagen Biosciences Inc., Guangzhou, China) (Figures 1(a) and 1(b)). The EF1*α* promoter-dependent lentiviral expression vector was used instead of the more potent and widely used CMV promoter because the EF1*α* promoter is less prone to silencing and provides more stable long-term expression. The single Tet-inducible lentiviral vector (pLV.TetIIP-GAL-EGFP-/Ubi-TetR), expressing rat galanin, and the reporter gene EGFP with the neomycin resistance gene under the control of the TetII promoter (*P*TetIIP) was constructed from TetR-based pLV.TetIIP-EGFP-/Ubi-TetR (GV347) lentiviral backbone vector system (Genechem Co. Ltd, Shanghai, China) (Figure 2). Briefly, GAL cDNA from the construct pBS KS(+)-GAL was obtained by PCR amplification using the following primer pairs: P1: 5′-AACCGTCAGATCGCACCGGCGCCACCATGGCCAGGGGCAGCGTTATCC-3′ and P2: 5′-TCACCATGGTGGCGACCGGGGACTGCTCTAGGTCTTCTG-3′ (418 bp). Subsequently, the cDNA was cloned into the linear expression vector GV347, and the transformants were identified by PCR using the primer pair and TetIIP-F: 5′-TGTCGA GGTAGGCGTGTA-3′ and EGFP-N-R: 5′-CGTCGCCGTCCAGCTCGACCAG-3′ (532 bp).

To obtain lentiviral particles, the 293 T packaging producer cells were cotransfected with the pLV/helper packaging plasmid mix (Invitrogen Life Technologies, USA) and the expression lentivector containing ExSi.P/Pgk-Puro, ExSi.P/Puro-EF1*α*-hTERT, TetIIP-EGFP−/Ubi-TetR, or TetIIP-GAL-EGFP−/Ubi-TetR plasmid, respectively, using Lipofectamine™ 2000 (Invitrogen Life Technologies, USA). After 48 h, the 293 T cells were lysed and replication-incompetent lentivirus was harvested. The lentivirus was filtered using a 0.45 mm pore size filter (Corning Inc., Corning, NY, USA) and concentrated approximately 1000-fold by ultracentrifugation (Beckman Coulter Inc., USA). The titers of infectious viral particles were determined by puromycin screening with crystal violet staining (Cyagen Biosciences Inc. Guangzhou, China). The viral stocks were aliquoted and stored at −80°C until further use.

### 2.3. Establishment and Identification of hTERT-Immortalized Rat BMSCs

Routine in vitro maintenance cultures were established using sterile frozen finite-lifespan Sprague Dawley (SD) rat BMSCs (RASMX-01001) (Cyagen Biosciences Inc., Guangzhou, China). The nature of these cells was confirmed based on positivity for CD90, CD29, and CD44 and negativity for CD34, CD11b, and CD45. The cells were cultured in a humidified incubator with 5% CO_2_ at 37°C in OriCell™ SD BMSC Growth Medium without a pH indicator (RASMX-90011, Cyagen Biosciences Inc.) and passaged (1 : 4) at 80% confluence. The medium was replaced at 3-day intervals. At passage 5 (P5), cultured rat BMSCs (5 × 10^4^ cells/mL) were seeded onto a 24-well dish. The next day, lentiviral particles (ExSi/Puro-EF1A-hTERT or ExSi/PGK-Puro) were added at a MOI (multiplicity of infection) of 20 in the presence of 4 *μ*g/mL polybrene (Sigma-Aldrich, St. Louis, MO, USA # 52495). After 8 h, the cells were washed and cultured with fresh medium containing 2 *μ*g/mL puromycin (Gibco, USA). After 2-3 weeks of selection, the surviving clones were isolated.

Following harvest of P10-transfected and P5-untransfected BMSCs, total RNA was isolated using TRIzol reagent (Invitrogen, USA). Reverse transcriptase polymerase chain reaction (RT-PCR) was performed using the following primer pairs for hTERT and the housekeeping gene GAPDH: 5-GGCTTCAAGGCTGGGAGGAAC-3 (forward) and 5′-A GCACACATGCGTGAAACCTG-3′ (reverse) for hTERT and 5′-CCTTCCGTGTTCCTA CCC -3′ (forward) and 5′-CAACCTGGTCCTCAGTGTAG-3′ (reverse) for GAPDH. The expected amplicon sizes for hTERT and GAPDH were 164 and 150 bp, respectively.

In order to detect the telomerase activity, total RNA was extracted from P11 BMSCs and P30 hTERT-BMSCs (10^5^-10^6^) lysates using the TRAPeze XL Telomerase Detection Kit (Chemicon, Temecula, CA, USA) according to the manufacturer's instructions. The fluorometric telomeric repeat amplification protocol (TRAP) was used to quantify telomerase activity. Briefly, the cell lysates were mixed with TRAPeze® XL reaction mix containing Amplifluor® primers and incubated at 30°C for 30 min. The amplified telomerase products were quantified using a fluorescence plate reader (Molecular Devices, LLC, Sunnyvale, CA, USA). Telomerase activity was subsequently calculated after comparing the ratio of telomerase products to an internal standard for each lysate (ΔFL/ΔR), and each sample was examined three times [22]. Human cervical carcinoma cells (HeLa cells) were detected as a positive control, whereas heat-inactivated cells (85°C) and ExSi/PGK-Puro-transduced BMSCs (PGK-BMSCs) were used as negative controls.

Cell proliferation was detected based on the incorporation of 5-ethynyl-29-deoxyuridine (EdU) using the EdU Cell Proliferation Assay Kit (Ribobio, Guangzhou, China). Briefly, P30 hTERT-BMSCs or P14 BMSCs were seeded onto 24-well plates and incubated with 25 *μ*M EdU for 24 h prior to fixation, permeabilization, and EdU staining according to the manufacturer's instructions. The cell nuclei were stained with Hoechst 33342 (Sigma-Aldrich, St. Louis, MO, USA) at a concentration of 10 *μ*g/mL for 10 min at room temperature. The proportion of cells that incorporated EdU was determined by fluorescence microscopy (Olympus, Tokyo, Japan). For growth curve analysis, P30 hTERT-BMSCs, P14 BMSCs, and P14 PGK-BMSCs were seeded onto 96-well plates with 2 × 10^3^ cells per well and grown in 200 *μ*L culture medium respectively. Over the following 7 days, cell proliferation was measured using an MTS assay (The CellTiter 96 kit, Promega, USA) in triplicate wells each day. The spectrophotometric absorbance of each sample was measured at 490 nm using a microplate reader (Bio-Rad Laboratories, Hercules, CA, USA), with blank control wells to zero absorbance. A standard curve was obtained to display the relationship between absorbance and cell numbers. A growth curve was drawn according to the standard curve [23]. The population doubling time was calculated as (PDT) = (log(*N*_*n*_/*N*_*n*−1_))/log2 at passage *n*, where *N* is the number of counted cells. Cell cycle assays were also performed in selected cells. Briefly, the trypsinized cells were fixed in 70% ethanol, washed with PBS, and then treated with 20 mg/mL RNase (TaKaRa, Otsu, Shiga, Japan) for 15 min at 37°C. DNA was labeled with 50 *μ*g/mL propidium iodide (PI; Sigma-Aldrich, St Louis, MO, USA) in the dark for 30 min at 4°C, and DNA content was assessed by flow cytometry with a Calibur (Becton Dickinson). Each group was analyzed in triplicate.

### 2.4. Phenotype and Neural Differentiation of hTERT-BMSCs

Surface molecules were detected using the monoclonal antibodies CD29, CD34, CD44, and CD45 (Becton Dickinson, USA) for the corresponding antigens of BMSCs by flow cytometry according to a standard protocol. In addition, to determine the neural differentiation of hTERT-BMSCs, the cells were removed from the flask bottom after the fourth passage, replated in 35 mm culture dishes, and induced after reaching 70–80% confluency in DMEM/F12 with B-27 supplement medium containing different epidermal growth factor (10 ng/mL) and basic fibroblast growth factor (20 ng/mL) for neuronal progenitor cell induction or glial-derived neurotrophic factor (10 ng/mL), brain-derived neurotrophic factor (10 ng/mL), and neurotrophin 3 (10 ng/mL) for neuron induction [24]. The shape of the induced cells was observed daily, and the differentiated cells were characterized by immunocytochemistry using neural-specific markers; undifferentiated hTERT-BMSCs cells were used as a control.

### 2.5. Tumorigenicity, Anchorage-Independent, and Karyotype Analysis

Like any genetic modification, cell immortalization may result in malignant transformation by impairing cell-cycle regulation. Thus, three four-week-old BALB/c nu/nu mice were subcutaneously injected with 0.1 mL of P40 hTERT-BMSC suspension each, and another 3 mice were injected with a human colon cancer SW480 cell suspension (ATCC, Manassas, VA, USA) as a positive control (5 × 10^6^ cells/mL each). Animals were maintained under sterile conditions for 4 months and palpated for tumor appearance once a week. To test for soft agar colony growth capacity, hTERT-immortalized cells were plated at a density of 1 × 10^5^ cells in 3 mL of 0.35% agarose over a 0.7% agar base in a 60 mm diameter culture dish. Cultures were fed every 3 days, and colonies with >50 cells were scored after 4 weeks in cultures under a dissecting microscope. Moreover, to determine if the abnormal karyotype resulted from ectopic hTERT, the cells were fixed using fresh stationary liquid (methanol : glacial acetic acid = 3 : 1), spread onto slides, and stained with Giemsa's solution, and the chromosome images were captured under an immersion objective using an Olympus BX51 High Class System Microscope (Olympus Corporation).

### 2.6. Determination of Tet-On Lentiviral Transfection Efficiency

The optimal transfection efficiency in the hTERT-BMSCs was determined based on the MOI and percentage of EGFP-positive cells using flow cytometry 2 days after LV.TetIIP-EGFP-/Ubi-TetR or LV.TetIIP-GAL-EGFP/Ubi-TetR transfection and doxycycline (Dox, Sigma-Aldrich, St. Louis, MO, USA) induction. The fraction of viral load to cell number was calculated as the MOI.

To directly observe the controllable gene expression, single Tet-on-inducible bicistronic lentiviral particles (LV.TetIIP-GAL-EGFP−/Ubi-TetR) expressing EGFP and GAL were used to infect hTERT-BMSCs, and viral supernatants at a MOI of 30 were added to fresh culture medium supplemented with 8 *μ*g/mL polybrene and 400 *μ*g/mL neomycin (Sigma-Aldrich, St. Louis, MO, USA). After 12 h, the cells were resuspended in fresh culture medium and then transferred to RPMI medium (Gibco, USA) containing 1 *μ*g/mL Dox (Sigma Aldrich, St. Louis, MO, USA) and supplemented with 10% Tet system-proved FCS (BD Biosciences, Clontech, USA), an optimal tetracycline-free serum for tetracycline-controllable expression systems. EGFP expression was observed under a fluorescence microscope after 48 h.

### 2.7. Detection of Inducible GAL Secretion from hTERT-BMSCs/Tet-on/GAL

Subconfluent hTERT-BMSCs were exposed to freshly filtered LV.TetIIP-GAL-EGFP-/Ubi-TetR viral supernatant at MOI 30 in the presence of 8 *μ*g/mL polybrene. After 12 h, the medium was replaced with fresh medium. At 48 h postinfection, the cells were subsequently placed in medium under neomycin selection (800 *μ*g/mL) for 10–14 days. The resulting neomycin-resistant cell clones were separated into cultures with and without Dox (0–10000 ng/mL) induction. A total of 16 positive clones were assessed using an EGFP fluorescence assay. Clone 4 was selected for its high induction of EGFP expression in response to Dox and low leakiness (activity in the absence of Dox) and subsequently named hTERT-BMSCs/Tet-on/GAL.

The kinetics of rat GAL protein levels secreted from neuronal differentiated hTERT-BMSCs/Tet-on/GAL under different concentrations of Dox was assayed using a galanin enzyme-linked immunosorbent assay (ELISA) kit (JM-E1001) according to the manufacturer's instructions (TSZ Biological Trade Co. Ltd, NJ, USA). Briefly, subconfluent neuronal differentiated hTERT-BMSCs/Tet-on/GAL (5 × 10^4^) were incubated in the presence of Dox at 0, 10, 100, 1000, 5000, and 10000 ng/mL for 48 h or incubated for various times in 12 h intervals with the administration of 1 *μ*g/mL Dox and removal of Dox. The supernatant from the cell culture medium was collected, centrifuged at 1000 rpm for 5 min at 4 °C, and subsequently incubated on a microplate precoated with a rat GAL monoclonal antibody for 45 min at 37°C (5 wells for each). After the second antibody conjugated with HRP was added and bound to the captured GAL, the HRP substrate TMB (tetramethylbenzidine) was added to the wells. The OD450 was measured to generate a standard curve and calculate the GAL concentration using a microplate reader (Molecular Devices, Sunnyvale, CA, USA). The secretion level was standardized and expressed in pg/mL of supernatant. hTERT-BMSCs were used as a control.

### 2.8. Statistical Analysis

Statistical analysis was performed using GraphPad Prism 5.01 for Windows (San Diego, CA, USA) via repeated measures one-way analysis of variance (ANOVA) followed by Bonferroni's multiple comparison test (telomerase activity) or two-way ANOVA followed by Bonferroni's posttests for evaluating the growth features and effects of Dox on GAL levels over time. Statistical significance was determined as *P* < 0.05. All data are presented as the mean ± standard error of the mean (SEM).

## 3. Results

### 3.1. Transcription of hTERT Gene and Telomerase Activity

**Figure 3:**
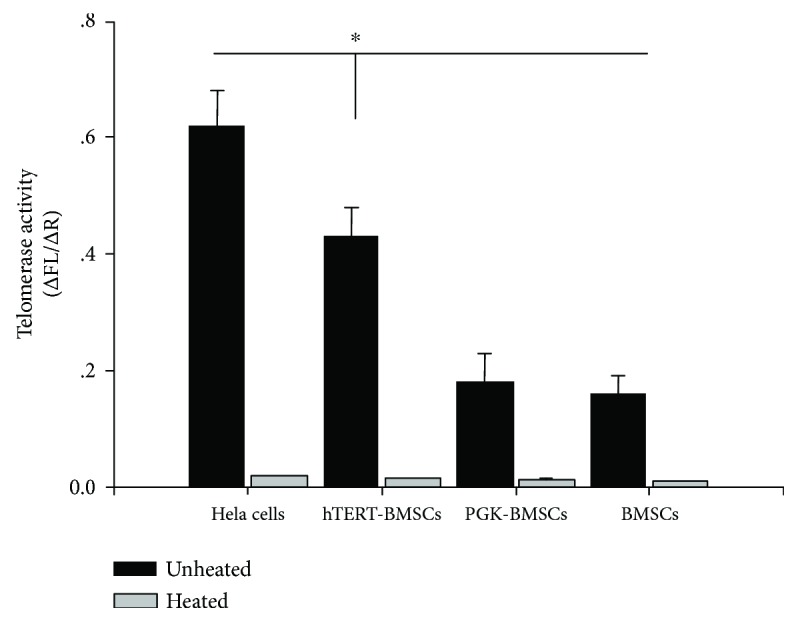
Telomerase activity of HeLa cells, BMSCs, PGK-BMSCs, and hTERT-BMSCs (TRAP assay) after disposed with or without heat. There was little telomerase activity in all heat-treated negative control cells; however, in unheated cells, despite extensive doublings, the immortalized cells (hTERT-BMSCs) displayed significantly higher telomerase activity than their primary counterparts and PGK-BMSCs, except the positive control cells (HeLa) and the telomerase activity of the immortalized population remained stable (assessed at P30). Significance level is *P* < 0.05, indicated by ∗.

mRNA expression of the hTERT gene was detected using RT-PCR with specific hTERT primers. For hTERT-transfected cells (hTERT-BMSCs), 164 bp-specific amplification bands of hTERT were detected, whereas for untransfected cells (BMSCs) and cells (PKG-BMSCs) transfected with the control vector, no specific band was detected (Supplemental information Figure S1(A)). Moreover, as internal reference fragments, a 150 bp-specific amplification band of GAPDH was detected in all cell groups (Supplemental information Figure S1(B)). These findings suggest that the hTERT gene was integrated into the genomic DNA of rat BMSCs and transcribed into mRNA. Subsequently, telomerase activity was also detected using TRAP for the sensitive measurement of telomerase activity. As shown in Figure 3, the immortalized population (hTERT-BMSCs) stably displayed higher telomerase activity (2.4 ± 0.5-fold) compared with their primary counterparts (BMSCs) and the negative control, even after extensive proliferation (up to 30 PDT), whereas much higher activity was observed in the positive control (HeLa cells). These results confirm the functionality of the implemented human telomerase gene in hTERT-BMSCs.

### 3.2. Growth Feature and EdU Proliferation Assay of hTERT-BMSCs

To confirm that rat BMSCs expressing hTERT had an extended lifespan, we monitored the growth features of the cells (i.e., hTERT-BMSCs expressing hTERT and untransfected BMSCs and PGK-BMSCs not expressing hTERT). The cell growth curves showed that immortalized BMSCs expressing hTERT vigorously proliferated for at least 6 days and subsequently reached a growth plateau due to inhibition of proliferation by cell-cell contact. In contrast, BMSCs and PGK-BMSCs without hTERT transduction grew slowly after plating and displayed growth retardation and senescence at 3-4 days after passage (Supplemental information Figure S2(A)). The PDT values for hTERT-BMSCs at P30, BMSCs, and PGK-BMSCs at P14 were approximately 25, 53, and 58 h, respectively. The results of flow cytometry revealed that most of the hTERT-transfected BMSCs at P30 showed a distinctive accumulation of G2/M and S phases, while, in striking contrast, untransfected or control PGK-BMSCs at P14 did not accumulate in G2/M and S phases, which implied most of cells ceased at senescence stage (see Supplementary Figure S2(B)).

**Figure 4:**
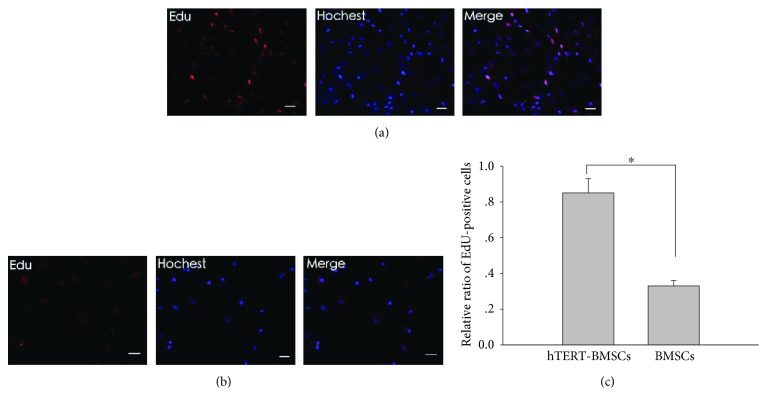
EdU proliferation assay of the effect of hTERT on the growth of BMSCs. The red fluorescent cells are in the S phase of mitosis, and the blue fluorescent cells represent all cells. (a) and (b) present images of P30 hTERT-BMSCs and P14 BMSCs, respectively. (c) Ratio of EdU-positive cells. Significance level is *P* < 0.05, indicated by ∗. Scale bar: 100 *μ*m.

To further assess the effects of hTERT on BMSCs, cell proliferation was also examined using an EdU assay, an immunochemical detection method that measures nucleotide analog incorporation into newly replicated DNA. Consistent with the results of the growth features, significantly more EdU-positive cells were observed among the hTERT-modified cells (Figures 4(a) and 4(b)), and the percentage of EdU-positive cells was significantly higher in P30 hTERT-BMSCs compared with P14 BMSCs (Figure 4(c)). These results further implied that ectogenic hTERT significantly lengthens the lifespan of cells, promoting DNA replication and telomere elongation and that hTERT immortality can favor cell proliferation. Thus, a stable hTERT-BMSC line with steady proliferation capacity was successfully generated.

### 3.3. Characterization of hTERT-BMSCs

**Figure 5:**
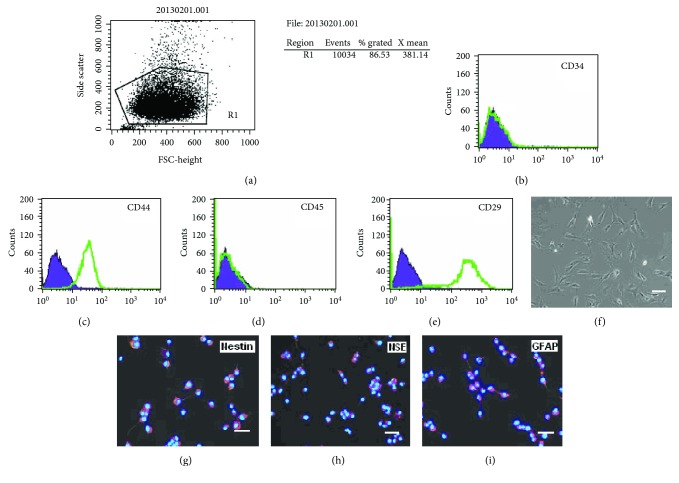
Identification of surface phenotype and neurogenesis of hTERT-BMSCs. (a–e) Expression of surface molecules detected using flow cytometry analysis. (f) Typical morphology was observed under an inverted phase contrast microscope. (g–i) The expression of neural specific markers on hTERT-immortalized BMSCs (note: nuclei were stained with Hoechst 33342 blue). Scale bar: 100 *μ*m.

In addition to becoming immortalized, the hTERT-BMSCs also retained the typical characterization of the parental cells (Figure 5). To assess cell phenotype, flow cytometry assay indicated that more than 85% of the hTERT-immortalized BMSCs were positive for typical surface markers of BMSCs (Figure 5(a)), including CD44 (85.08%) and CD29 (95.16%), whereas these cells were almost completely negative for both hematopoietic markers CD34 (0.21%) and CD45 (0.09%) (Figures 5(b)–5(e)). The cells retained a phenotype identical to that of their primary counterparts (Figure 5(f)). To assess neurogenic differentiation, the isolated hTERT-BMSCs were cultured in neuronal induction medium. Although the number of cells did not increase, the development of axon-like and dendrite-like cells indicated neuronal differentiation. To further confirm that the differentiated cells were neurogenic, the differentiated cells were detected with specific neural markers. Immunofluorescence assays demonstrated that the cells were positive for the expression of Nestin, a marker of neuronal progenitor cells, NSE, a marker of neurons, and GFAP, a marker of glial cells (Figures 5(g)–5(i)), while undifferentiated hTERT-BMSCs showed no marked expression of neural markers (data not shown).

The tumorigenicity of the hTERT-immortalized BMSC line was subsequently investigated in vivo after subcutaneous injection in the flanks of nude mice. After 2 months postinjection, the nude mice injected with SW480 cells developed tumors; in contrast, malignant transformation did not occur in mice injected with hTERT-BMSCs for up to 4 months and no colony growth in soft agar was found (data not shown). Moreover, karyotype analysis revealed that hTERT-BMSCs displayed a chromosomal pattern similar to that of the parental cells (diploid number 42), with no abnormal nuclear pattern observed after genetic modification (Supplemental information Figure S3), thus demonstrating the homogeneity and safety of the generated cell line.

### 3.4. Construction and Transfection Efficiency of Single Tet-Inducible Lentiviral Vector

To establish a Dox-dependent GAL gene expression system with a single lentiviral vector, we inserted the expression cassettes containing the rat GAL cDNA fragment (Supplemental information Figure S4A) from the construct pBS KS(+)-GAL into the single Tet-regulated lentiviral vector system “GV347” (Figure 2). Transformants of the cloned insert were subsequently identified by PCR and electrophoresis on a 1% agarose gel. Five positive transformants with the proper orientation were detected (Supplemental information Figure S4(B)), and the sequences were confirmed by DNA sequencing.

The fraction of cells that glowed green, reflecting lentiviral gene transfer efficiency, increased dose dependently as the MOI increased from 0 to 30. The highest infection rate, approximately 78%, was obtained at an MOI of 30, and no significant increase was observed when the amount of virus increased from 30 to 75 MOI. In addition, the number of dead cells floating in the medium significantly increased. Therefore, an MOI of 30 was used in subsequent experiments.

### 3.5. Visualization of EGFP Fluorescence under the Dox Induction

**Figure 6:**
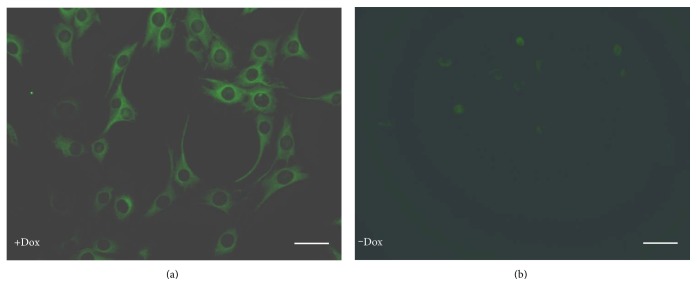
Regulatable EGFP expression from hTERT-BMSCs/Tet-on/GAL cells in the presence and absence of Dox in vitro (×400). hTERT-BMSCs transfected with LV.TetIIP-GAL-EGFP-/Ubi-TetR showed strong green fluorescence after treatment with 1 *μ*g/mL Dox for 48 h (a), whereas very faint fluorescence was observed without Dox induction (b). Scale bar: 200 *μ*m.

The expression of EGFP in response to Dox was directly observed in the hTERT-BMSCs transfected with LV.TetIIP-GAL-EGFP-/Ubi-TetR (Figure 2). The expression of EGFP was induced using a Dox at concentrations of 1 *μ*g/mL, whereas untreated cells (without Dox) exhibited little green fluorescence (Figures 6(a) and 6(b)). These results suggested that the functional tetracycline-controlled transgene can effectively be delivered with higher inducibility into mammalian cells using the developed single Tet-on vector system.

### 3.6. Inducible GAL Secretion from hTERT-BMSCs/Tet-on/GAL

**Figure 7:**
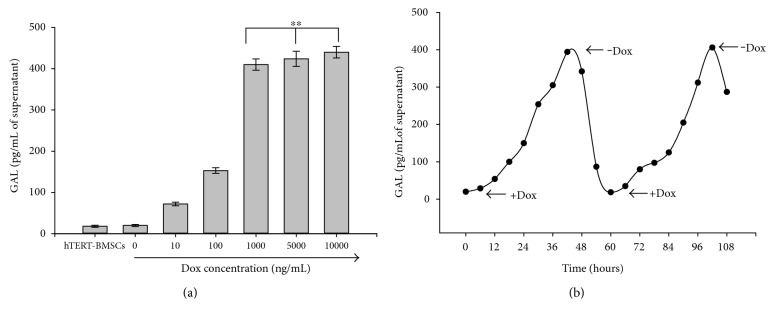
GAL secretion from cells after Dox induction in vitro. (a) The dose response of galanin production in cultured hTERT-BMSCs/Tet-on/GAL revealed a correlation between the Dox dose and the galanin secretion level. Significance level is ^∗∗^*P* > 0.05, indicated by ∗∗. (b) Time course of galanin secretion from hTERT-BMSCs/Tet-on/GAL under the control of Dox. The arrows indicate the addition (+Dox) or removal (−Dox) of Dox from the culture medium.

The inducible secretion of rat GAL from hTERT-BMSCs/Tet-on/GAL by Dox after neuronal differentiation was initially confirmed by ELISA using hTERT-BMSCs as negative controls. After treatment for 48 h with different concentrations of Dox (Figure 7), low endogenous GAL secretion (20 pg/mL of supernatant) was observed in the parental hTERT-BMSCs. However, the secretion of GAL from hTERT-BMSCs/Tet-on/GAL increased in response to treatment with an increased concentration of Dox greater than 10 ng/mL in the culture medium, peaking under Dox induction at 1000 ng/mL (approximately 400 pg/mL of supernatant) (Figure 7(a)). We also assessed the reversibility of Dox induction after subjecting the hTERT-BMSCs/Tet-on/GAL to repeated on-off cycles. When Dox was added to the culture medium (1 *μ*g/mL), GAL secretion gradually increased to 20-fold after 48 h and subsequently returned to basal levels after Dox removal for approximately 12 h. Comparable results were obtained during the second on-off cycle (Figure 7(b)). These results suggest that GAL gene expression from these genetically modified cells (hTERT-BMSCs/Tet-on/GAL) can be regulated by the addition of Dox in vitro.

## 4. Discussion

To explore a novel strategy for establishing controllable expression of the exogenous GAL gene from hTERT-BMSCs for pain therapy, we constructed an hTERT-BMSCs/Tet-on/GAL line using a single Tet-on-inducible lentiviral vector. This cell line displayed low baseline activity coupled with high inducibility in the presence of low doses of the inducer Dox in vitro.

The management of neuropathic pain after nerve injury remains a major clinical challenge. The combination of ex vivo gene transfer and cell transplantation is considered a potentially useful strategy for the treatment of neurodegenerative diseases and traumatic injuries. This approach requires optimized gene delivery systems for therapeutic molecules. Several cellular vehicles have been investigated for ex vivo gene therapy of the CNS [25], and attention has focused on the therapeutic potential of BMSCs from bone marrow. BMSCs do not induce an allogenic reaction and might even suppress host T cell proliferation, suggesting that cells cultured from a single donor might be expanded to form a reserve pool that could be used for multiple recipients [26]. However, during in vitro culture, BMSCs undergo replicative senescence and lose the ability to proliferate over time, as observed in the present study. This decline has been attributed to genetic instability after critical shortening of telomeres. Lentiviral vectors have been used for the stable integration and long-term expression of transgenes. Lentiviral vectors are also favorable for biological research and gene therapy trials due to their ability to infect both dividing and nondividing cells and are particularly suitable for BMSCs [27]. Thus, in the present study, using a lentiviral system, we introduced the immortalization gene, hTERT, into primary rat BMSCs. Untransfected BMSCs without hTERT expression exhibited reduced growth associated with aging in vitro and failed to proliferate, becoming senescent after passage 14. In contrast, after transduction with hTERT, hTERT-immortalized BMSCs exhibited strong proliferation. The PDT of hTERT-BMSCs is shorter than that of nonimmortalized cells. After more than 30 passages, hTERT-BMSCs retain the potential to divide further. Similarly, significantly more EdU-positive cells were observed among the cells with ectogenic hTERT expression, indicating that hTERT-BMSCs maintained higher telomerase activity and proliferated significantly longer than wild type BMSCs. Moreover, the successive genetic modifications and extensive proliferation of these cells did not lead to an alteration of the mesenchymal phenotype, as assessed using conventional markers. The phenotype and karyotype of hTERT-BMSCs did not differ significantly from those of untransfected cells. Moreover, these cells retained the normal morphology and neuronal differentiation characteristics of stem cells when cultured in induction media. Based on the negative results of colony growth in soft agar and the lack of tumorigenicity in nude mice, our data clearly indicated that the hTERT-immortalized BMSCs did not exhibit any neoplastic transformation phenotype at least up to 4 months. Although telomerase expression is a hallmark of cancer and spontaneous tumoral transformation of MSCs expressing hTERT was reported [28], telomerase overexpression is typically nononcogenic, and hTERT-transduction has not hitherto been associated with neoplastic transformation. Many previous studies have investigated the long-term effects of forced expression of human telomerase catalytic component in normal human fibroblasts or BMSC. In vitro growth requirements, cell cycle checkpoints and karyotypic stability in telomerase-expressing cells are similar to those of untransfected controls. In addition, coexpression of telomerase, the viral oncoproteins HPV16 E6/E7 (which inactivate p53 and pRB), and oncogenic Ras does not result in growth in soft agar. Thus, although ectopic expression of telomerase in primary cells is sufficient for immortalization, it does not result in changes typically associated with malignant transformation that could be served as stem cell-based vehicles for therapeutic gene delivery in the CNS [29, 30]. But, even so, the possibility of transformation which occurs after long-term expansion of hTERT-BMSCs still need further study.

Because of the relationship of the GAL gene to galanin production and the activation of galanin through central GAL receptors in the CNS [31], we introduced the GAL gene into hTERT-BMSCs as a potential treatment for chronic neuropathic pain. Although previous studies have demonstrated that IAST genetically modified by the rat preprogalanin gene secretes higher levels of galanin in vitro and efficiently functions to relieve neuropathic pain [8], potential complications resulting from the continuous secretion of GAL appear inevitable. In most cases, the successful application of gene therapy requires the development of vectors that permit regulated control of therapeutic gene expression. Tet-regulatable systems have been successfully used to regulate transgene expression in established cell lines and transgenic animals. There are two basic variants of the tetracycline-inducible expression system: the tTA (Tet-off) system and the rtTA (Tet-on) system [32]. Typically, if a gene remains predominantly inactive and is only occasionally activated, then the Tet-on system is more appropriate than the Tet-off system. Leaky expression due to both inherent defects in Tet-based systems and promoter leakiness resulted from promoter-dependent or integration site-dependent effects compromises the desired stringent regulation of transgene expression [33]. Thus, a variety of methods for integrating Tet-inducible expression components into a single vector has recently been described to obviate the selection of a homogeneous and cotransduced population [34, 35]. In consideration of most doxycycline-responsive systems that are based on the TetR-VP16 chymeras, in these systems, the promoter is only active when the tTA or the rtTA transactivators bind the regulated promoter; the expression of the VP16 transactivators in the regulated cells can have several undesired consequences such as alteration of the promoter natural activity, activation of cellular genes, and toxicity [36]. To this end, we engineered a convenient TetR-based inducible transgene expression vector as a single Tet-inducible bicistronic lentivirus system to regulate EGFP and GAL expression in a single vector, in which EGFP under the control of the TetII promoter was used as a reporter. This system is less toxic than the rtTA since it does not have the transactivator VP16. In the present study, the inducibility and background EGFP expression of recombinant Tet-on lentiviral particles were determined after transfecting hTERT-BMSCs at an MOI of 30. As shown in Figure 6, the basal expression of EGFP was nearly absent from transfected hTERT-BMSCs without Dox, whereas treatment with Dox (1 *μ*g/mL) resulted in marked expression of EGFP.

Although the expression of transgenes (often EGFP) can be regulated in stem cells and their differentiated progenies at an early stage, transgenes are frequently no longer expressed or regulated in mature cells, including neurons [37]. In our present study, a low level of GAL secretion from hTERT-BMSCs/Tet-on/GAL was detected in vitro in the noninduced state after neuronal differentiation, whereas an extremely low level of Dox induction (10 ng/mL) was able to activate GAL expression (see Figure 7(a)). The level of GAL secretion was controlled by Dox in an apparently dose-dependent manner, with a positive linear trend in GAL production with respect to the inducer. Additionally, as shown in Figure 7(b), kinetic tests revealed that GAL gene activation could be repeated in on-off-on cycles, and long-term-regulated GAL expression was achieved by daily administration of Dox. These observations indicate that the Dox-based regulation of GAL secretion from hTERT-BMSCs/Tet-on/GAL was controllable, rapidly reversible, and not lost over time. Transgene regulation was also achieved in mature neurons and astrocytes after differentiation in vitro, suggesting the potential for effective function after transplantation into the CNS.

### 4.1. Study Limitations

Despite of significant differences in GAL secretion from transfected cells observed in the presence and absence of Dox, the weak leakage of galanin protein is problematic, and EGFP expression from hTERT-BMSCs/Tet-on/GAL was still observed in ELISA and fluorescence assays even in the off state, reflecting the unexpected translation in transduced cells in the absence of Dox [38]. Thus, the Tet-on system used in the present study still has specific limitations. Fortunately, the leaky expression of the transgenes was insignificant compared with the expression in transduced cells exposed to Dox. Therefore, unless the transgenes can affect cellular processes at an extremely low level, these vectors should be suitable for most target genes in biological research. However, an improved promoter other than TRE would enable the further reduction or even elimination of leakiness in this inducible system. Moreover, the differences in basal expression between cell lines might reflect the location of the insertion within the host genome and the influence of surrounding host genes on target gene expression. Therefore, this basal level of expression could be decreased by advanced screening for BMSCs line with low levels of expression in the off state. Thus, additional studies are needed to improve the system.

In summary, using a single tetracycline-inducible lentivirus delivery system to introduce the therapeutic GAL gene into rat hTERT-immortalized BMSCs, we generated an hTERT-BMSCs/Tet-on/GAL cell line with inducible rat GAL expression to regulate the secretion levels of GAL in vivo, which is critical for the balance of excitatory and inhibitory systems in pain-processing centers. This strategy is the first promising step toward a novel stem cell-based “biological mini pump” for potential use in pain therapy.

## Supplementary Material

Legends of Supplemental Figures. Figure S1. RT-PCR for hTERT mRNA expression from cells *in vitro*. The up-regulated expression of hTERT mRNA in BMSCs transfected with pLV.ExSi/Puro-EF1α-hTERT compared with BMSCs transfected with empty vector pLV.ExSi/PGK-Puro or BMSCs without transfection. BMSCs, PGK-BMSCs, hTERT-BMSCs and DL–1000 DNA ladder (lanes 1–4). GAPDH was used as an internal reference. Figure S2. Growth and proliferation feature assay of hTERT-BMSCs. (A) Growth curvesof BMSCs, PGK-BMSCs and hTERT-BMSCs. (B) Cell cycle distribution of BMSCs and BMSCs transfected with control or hTERT. Significance level is *P* < 0.05, indicated by ∗. Figure S3. Karyotype analysis of hTERT-BMSCs. hTERT-BMSCs displayed the same chromosomal pattern as their parental cells (diploid number 42) after genetic modification to express exogenous hTERT. Figure S4. Identification of rat GAL cDNA (A) and recombinant transformants (B) by PCR.
