# Supplementary material for: hTERT-Immortalized Bone Mesenchymal Stromal Cells Expressing Rat Galanin via a Single Tetracycline-Inducible Lentivirus System
Source: Stem Cells Int. 2017 May 11;2017:6082684. doi: 10.1155/2017/6082684 (PMC5444038; doi:10.1155/2017/6082684)
Supplement: Supplementary file 1 — Legends of Supplemental Figures. Figure S1. RT-PCR for hTERT mRNA expression from cells in vitro. The up-regulated expression of hTERT mRNA in BMSCs transfected with pLV.ExSi/Puro-EF1α-hTERT compared with BMSCs transfected with empty vector pLV.ExSi/PGK-Puro or BMSCs without transfection. BMSCs, PGK-BMSCs, hTERT-BMSCs and DL–1000 DNA ladder (lanes 1–4). GAPDH was used as an internal reference. Figure S2. Growth and proliferation feature assay of hTERT-BMSCs. (A) Growth curvesof BMSCs, PGK-BMSCs and hTERT-BMSCs. (B) Cell cycle distribution of BMSCs and BMSCs transfected with control or hTERT. Significance level is P < 0.05, indicated by ∗. Figure S3. Karyotype analysis of hTERT-BMSCs. hTERT-BMSCs displayed the same chromosomal pattern as their parental cells (diploid number 42) after genetic modification to express exogenous hTERT. Figure S4. Identification of rat GAL cDNA (A) and recombinant transformants (B) by PCR. [file 6082684.f1.doc]

**Appendix: Supplementary Information**

**Legends of Supplemental Figures**

**Figure S1**. RT-PCR for hTERT mRNA expression from cells *in vitro.* The up-regulated expression of hTERT mRNA in BMSCs transfected with pLV.ExSi/Puro-EF1α-hTERT compared with BMSCs transfected with empty vector pLV.ExSi/PGK-Puro or BMSCs without transfection. BMSCs, PGK-BMSCs, hTERT-BMSCs and DL-1000 DNA ladder (lanes 1–4). GAPDH was used as an internal reference.

**Figure S2.** Growth and proliferation feature assay of hTERT-BMSCs. (A) Growth curves of BMSCs, PGK-BMSCs and hTERT-BMSCs. (B) Cell cycle distribution of BMSCs and BMSCs transfected with control or hTERT. Significance level is *P* < 0.05, indicated by *.

**Figure S3.** Karyotype analysis of hTERT-BMSCs. hTERT-BMSCs displayed the same chromosomal pattern as their parental cells (diploid number 42) after genetic modification to express exogenous hTERT.

**Figure S4.** Identification of rat GAL cDNA (A) and recombinant transformants (B) by PCR.

**Supplemental Figures**

**Fig. S1**

**
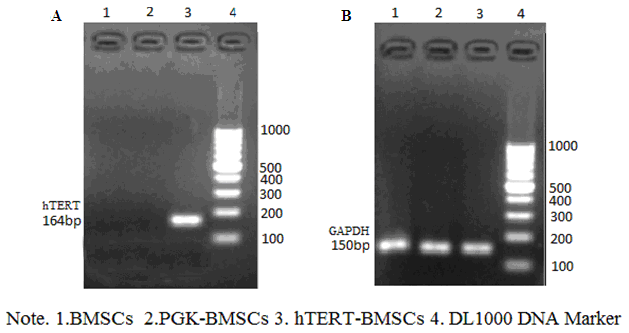
**

**Fig. S2**

**A**

**B**

**
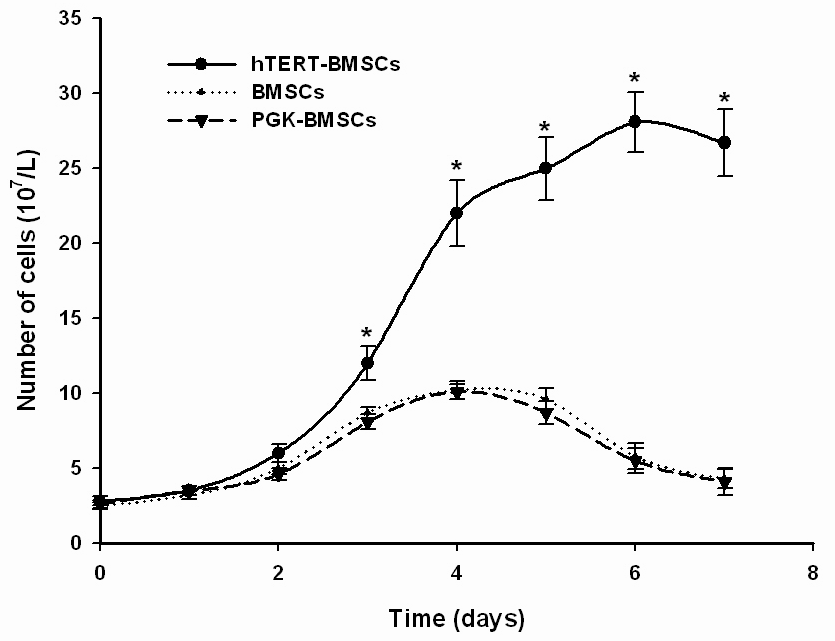

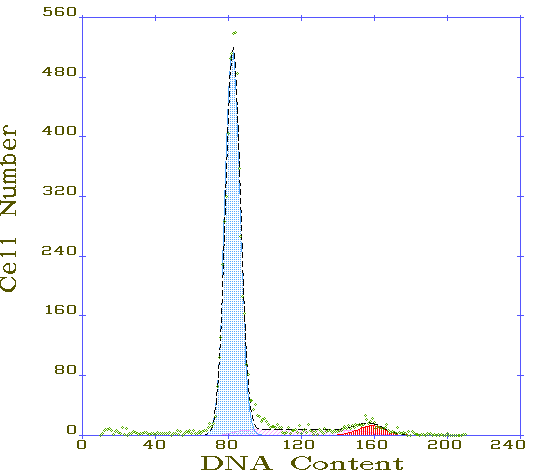

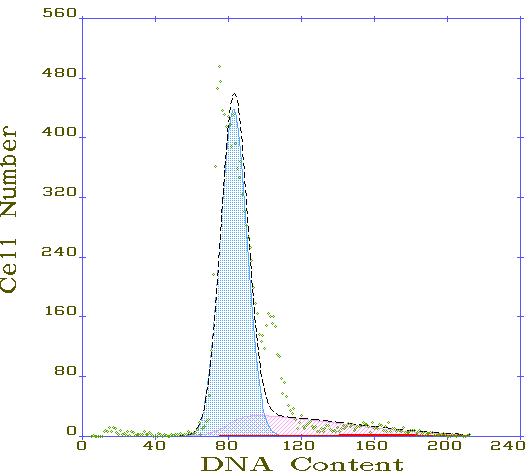

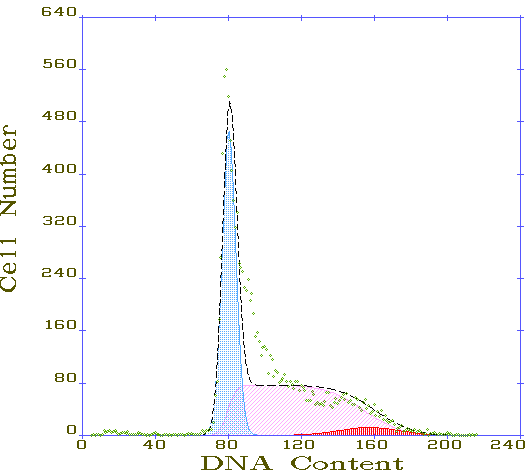
**

**BMSCs**

**PGK-BMSCs**

**hTERT-BMSCs**

|  | **G_0_/G_1_** | **S** | **G_2_/M** | **S+G_2_/M** |
| --- | --- | --- | --- | --- |
| **BMSCs**  **PGK-BMSCs**  **hTERT-BMSCs** | **85.07±1.16**  **84.22±1.42**  **68.33±0.93^*^** | **12.47±2.05**  **13.81±1.94**  **26.50±1.85^*^** | **2.50±0.90**  **2.09±0.99**  **5.20±0.97^*^** | **14.97±1.16**  **15.90±0.94**  **31.70±0.90^*^** |

**Fig. S3**


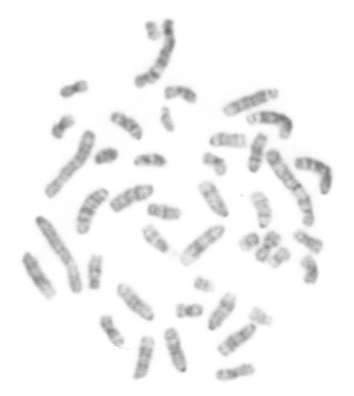

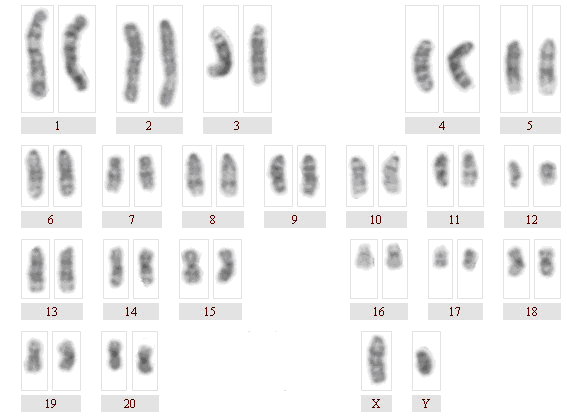


**Fig. S4**

**
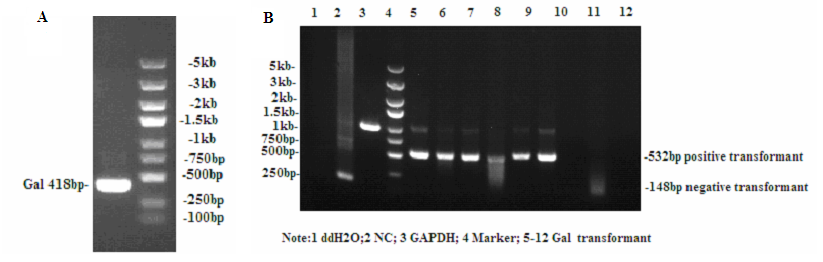
**
